# *Nocardia* Infection Presented as Intramuscular Abscess in a Kidney Transplant Recipient: Case Report and Literature Review

**DOI:** 10.1155/crin/9102520

**Published:** 2025-10-28

**Authors:** Ghia Spangenberg, Syme Aftab, Rajiv Juneja, David L. Gordon, Jordan Y. Z. Li

**Affiliations:** ^1^Renal Unit, Flinders Medical Centre, Bedford Park, Adelaide 5042, South Australia, Australia; ^2^College of Medicine and Public Health, Flinders University, Bedford Park, Adelaide 5042, South Australia, Australia; ^3^Department of Microbiology and Infectious Diseases, Flinders Medical Centre, Bedford Park, Adelaide 5042, South Australia, Australia

**Keywords:** immunosuppression, intramuscular abscess, kidney transplant, *Nocardia pseudobrasiliensis*

## Abstract

**Background:**

Nocardia is a gram-positive bacterium capable of causing both local and systemic infections, typically in immunocompromised patients. The most common clinical presentation is pulmonary infection. Muscle abscess due to Nocardia infection without disseminated nocardiosis is rare.

**Case Report:**

In this report, we describe the case of a 67-year-old male kidney transplant recipient who developed a swelling of the right biceps muscle. CT imaging showed an intramuscular abscess, which was subsequently surgically drained. The drained fluid grew *Nocardia pseudobrasiliensis*, which was resistant to imipenem but susceptible to trimethoprim/sulfamethoxazole (TMP-SMX), ciprofloxacin and linezolid. The patient was initially treated with TMP-SMX and ciprofloxacin. Treatment was changed to 6 months of ciprofloxacin and azithromycin due to intolerance to TMP-SMX which resulted in a good clinical response and no recurrence for 3 years. We also review all previously reported *N. pseudobrasiliensis* infections and cases of nocardiosis presenting as intramuscular infections.

**Conclusion:**

Given its prevalence amongst immunocompromised patients, Nocardiosis requires consideration in the differential diagnosis for the cause of atypical infections in transplant recipients. The antimicrobial susceptibilities of *Nocardia* are variable depending on species. Of key note, carbapenem resistance has been recently described in *N. pseudobrasiliensis*. This development should be considered when initiating antimicrobial therapy to ensure good patient response to treatment.

## 1. Introduction

*Nocardia* spp. are Gram-positive bacteria capable of causing both local and systemic infections. Although nocardiosis has been traditionally viewed as an opportunistic infection, immunocompetent subjects may also be infected. However, disease in immunocompromised patients tends to be more severe and disseminated [1]. *Nocardia pseudobrasiliensis* is a relatively newer species. It is distinguishable from *N. brasiliensis* given its distinctions in disease patterns and antibiotic susceptibility profiles. In particular, the former is more strongly associated with invasive and disseminated disease [1].

We report an unusual case of a nocardiosis presenting as a localised intramuscular abscess caused by *N. pseudobrasiliensis* in a renal transplant recipient.

## 2. Case Report

A 67-year-old man with end-stage kidney disease secondary to autosomal dominant polycystic kidney disease received a deceased-donor kidney transplant three years prior. His renal transplant was complicated by an episode of cytomegalovirus viraemia in the early post-transplant period which was treated with valganciclovir. Induction immunosuppressive therapy included antithymocyte globulin (ATG). His immunosuppressive regimen included tacrolimus and prednisolone. Mycophenolate was ceased due to significant leukopenia. His allograft function was stable with serum creatinine of 130 μmol/L (60–110 μmol/L).

His other comorbidities include type II diabetes mellitus, hypertension, gout, a previous posterior circulation embolic cerebrovascular accident and recent unproved pulmonary embolism for which he has been anticoagulated with apixaban.

Given his good allograft function, his left arteriovenous fistula was ligated 12 months post-kidney transplant. Swelling of the opposite right biceps brachii was noted 2 days after this procedure in the vicinity of an intravenous cannula inserted at the time of fistula ligation. Ultrasound showed a 10 × 20 × 20 mm intramuscular fluid collection presumed to be an intramuscular haematoma given the recent commencement of apixaban. The patient represented to hospital a few days later in the setting of increasing pain and swelling of the right biceps. Physical examination did not reveal a palpable mass. There were no accompanying constitutional symptoms and the patient was apyrexial. Repeat ultrasound showed a persistent heterogenous hypoechoic collection now measuring 71 × 48 × 47 mm. Computed tomography (CT) confirmed a deep collection in the right biceps with low density centrally and rim enhancement on delayed phase suggestive of an intramuscular abscess.

Laboratory investigations performed at this time showed a haemoglobin of 141 g/L (reference range 135–175 g/L) and white cell count of 4.86 × 10^9^/L (4–11 × 10^9^/L) with an absolute neutrophil count of 3.26 × 10^9^/L (1.80–7.5 × 10^9^/L). C-reactive protein was 146 mg/L (0.0–8.0 mg/L), and serum creatinine was 109 µmol/L (60–110 μmol/L). The tacrolimus level was 6.3 μg/L (5–15 μg/L).

Subsequent incision and drainage were performed with the intraoperative finding of a large collection of pus within the right bicep muscle. Microscopy of the intraoperative sample showed numerous polymorphonuclear leukocytes and branching Gram-positive bacilli resembling *Nocardia*. Modified Ziehl–Neelsen stain was positive. Empiric treatment included meropenem and linezolid. Species identification was confirmed as *N. pseudobrasiliensis* by matrix-assisted laser desorption/ionization time-of-flight mass spectrometry (MALDI-TOF MS) with Version 10 of the Bruker database (Bruker Daltonics, Bremen, Germany). 16S rRNA gene sequencing and BLAST analysis showed 100% identity with *N. pseudobrasiliensis* type strains ATCC 51512 and DSM 44290. Susceptibility testing was performed by broth microdilution and showed characteristic resistance to imipenem and susceptibility to ciprofloxacin, SXT, amikacin, clarithromycin and linezolid. Blood cultures were negative.

Whole body CT showed an area of consolidation in the right middle lobe but no other foci of infection. The CT findings were present on previous imaging of the chest but were thought to represent a pulmonary infarct rather than infection given the appearance and the known occurrence of a recent pulmonary embolism. Magnetic resonance imaging (MRI) of the brain was also performed, showing no evidence of cerebral abscess.

The treatment regimen was subsequently changed to linezolid and trimethoprim/sulfamethoxazole (TMP-SMX). On discharge from hospital, the antibiotic regimen was further revised to oral ciprofloxacin and TMP-SMX due to the concern of linezolid causing bone marrow suppression. TMP-SMX was subsequently ceased due to persistent hyperkalaemia and replaced with azithromycin. Treatment with ciprofloxacin and azithromycin was given for 6 months with good response.

A repeat CT scan of the chest 2 months into treatment showed resolution of the right middle lobe consolidation. There has been no evidence of recurrence 30 months after ceasing antimicrobials.

## 3. Discussion

Nocardiosis is a rare bacterial infection which can affect immunocompetent individuals, but more frequently causes disease in immunocompromised patients, with glucocorticoid therapy as the leading risk factor [2]. Nocardiosis is caused by aerobic actinomycetes which are found in soil and putrefying plant matter, with infection typically caused by inhalation or direct inoculation [3–5]. *Nocardia* spp. cause both localised and systemic infections, with the most frequent clinical presentation being pulmonary infection [5]. Target organs affected by disseminated nocardiosis most commonly include the central nervous system (CNS) and cutaneous tissues [6]. *N. asteroides* is the most common organism within the genus to cause disease, followed by *N. brasiliensis* [7, 8].

Infection in our case was caused by *N. pseudobrasiliensis*. This organism was first identified in 1996, with 16S rRNA gene sequence analysis and DNA-DNA hybridisation confirming that this taxon constituted a new species [8]. However, to date, few cases have been reported [7–9]. *N. pseudobrasiliensis* differs from *N. brasiliensis* by way of its adenine metabolism and nitrate reductase activity [7]. The species further differ in terms of clinical presentation; *N. pseudobrasiliensis* typically causes invasive and disseminated disease as opposed to localised disease, which is more commonly associated with *N. brasiliensis* [10]. Moreover, *N. pseudobrasiliensis* also causes disease in immunocompetent patients [10]. Importantly, the species differ in their susceptibility profiles, with *N. pseudobrasiliensis* showing variable susceptibility to carbapenems, while *N. brasiliensis* is inherently resistant to ciprofloxacin [10, 11].

Extensive search of the literature revealed 17 cases (Table 1) of *N. pseudobrasiliensis,* with only one other case of an intramuscular abscess [8]. This case involved a 79-year-old man with bilateral multifocal intramuscular leg abscesses in the setting of long-term steroid use for myasthenia gravis, as well as pulmonary infection with the abscesses presumed to be caused by dissemination [8]. This finding raises the possibility of a similar dissemination mechanism in our patient. After treatment with linezolid for less than 3 weeks, followed by moxifloxacin for 6 months, there was no recurrence at 1-year follow-up.

A further search of *Nocardia* presentations with intramuscular involvement yielded 23 cases in the English language, including the present case, as outlined in Table 2. Of these, *N. farcinica* was the most frequent causative organism, occurring in 9 cases [4, 23–25, 32, 35–38], followed by *N. asteroides* in 4 cases [30, 33, 34, 39]. *N. pseudobrasiliensis* presented twice [8], including our case. Leg muscles, particularly involving the thigh, were most frequently affected, with back, shoulder and upper limb muscle abscesses presenting less frequently. This places our case in a rarer context, given it was the biceps brachii that was affected. Multiorgan involvement, usually of the lungs and brain, occurred in almost every case.

While immunosuppression was a leading risk factor, 30% did not report an immunosuppressive risk, suggesting that a *Nocardia* differential diagnosis should not be neglected in immunocompetent patients. Surgical intervention was required in 17 cases, with a minority resolving solely on medical treatment. Surgical drainage was the primary approach, as also seen in our case, although debridement was necessary in 4 cases. Concerning antibiotic choice, TMP-SMX has been recommended as the first-line agent [41, 42] and was commonly utilised, as seen in 18 cases. However, given that dual coverage is recommended in complex cases, especially with CNS involvement [42], a combination of antibiotics was given to most patients, with agents including imipenem, meropenem, amikacin, linezolid, ciprofloxacin and ceftriaxone demonstrating effectiveness by either improving or resolving infection-related symptoms. Both our case and that of Veerappan Kandasamy et al. [8] featured nocardiosis due to *N. pseudobrasiliensis* with intramuscular involvement. Here, linezolid was an initial shared antibiotic. However, a key difference to note was that TMP-SMX was administered to our patient, before being substituted for azithromycin, with the patient's symptoms fully resolving with no recurrence to date.

Nocardiosis most commonly occurs in patients with malignancy, solid organ transplant, human immunodeficiency virus infection and long-term corticosteroid use [2, 43]. Regarding the solid organ transplant population, *Nocardia* is responsible for 0.7%–3% of infections, predominantly in kidney, heart and liver transplant recipients [2]. Additional risk factors include supratherapeutic calcineurin inhibitor levels and prior cytomegalovirus infection [2, 43, 44]. Our case had a history of cytomegalovirus viraemia; however, not in the preceding 6 months, which was the timeframe the literature has demonstrated [2, 43]. The other known case of *N. pseudobrasiliensis* in a renal transplant recipient was a 68-year-old man with pulmonary infection in the setting of supratherapeutic tacrolimus levels [17]. This patient's elevated mean calcineurin inhibitor levels in the 30 days prior were a risk factor for developing *Nocardia* infection [2].

Nocardiosis can be treated successfully with prolonged courses of sulfonamides, particularly for localised disease in combination with surgical drainage or debridement [45]. However, TMP-SMX, which is commonly prescribed for *Pneumocystis jirovecii* prophylaxis in transplant recipients, may not be sufficient to prevent Nocardia infection, and resistance has also been reported amongst *N. pseudobrasiliensis* isolates [46]. Disseminated disease in immunocompromised patients generally warrants a two-drug treatment regimen given the high mortality associated with Nocardia infection in these patients, the diversity of *Nocardia* species and their variable antibiotic susceptibility profiles [45]. Schlaberg et al. report linezolid as an effective agent against various nocardial strains, including *N. pseudobrasiliensis,* particularly given its extensive bioavailability and tissue distribution [46], Linezolid has been utilised in numerous Nocardia infections with intramuscular involvement as seen in Table 2. Moreover, given its observed effectiveness against *N. pseudobrasiliensis,* fluoroquinolones have also been recommended [8], as further corroborated by our case. Clarithromycin or azithromycin and tobramycin have also demonstrated efficacy against *N. pseudobrasiliensis* [46]. While these cases point towards 4–6 months of therapy, the optimal duration of antibiotic therapy currently remains elusive. This should be addressed in future studies to mitigate the risk of under- and overprescribing antibiotics, both of which are associated with suboptimal clinical outcomes, especially in the context of multidrug-resistant strains of *N. pseudobrasiliensis.*

## 4. Conclusion

Nocardiosis requires consideration in the differential diagnosis of infections in immunocompromised patients especially with unusual presentations. This is particularly critical given the speciation, clinically relevant differences in disease patterns and antibiotic susceptibility between *N. pseudobrasiliensis* and *N. brasiliensis*. A multidrug antibiotic course for systemic infection alongside surgical drainage for the local manifestations proved to be an effective treatment approach. Nevertheless, evidence supporting a wider range of potential treatments needs to be explored, with continued investigation of optimal treatment regimens for patient outcomes especially in the context of multidrug-resistant strains of *N. pseudobrasiliensis.*

## Figures and Tables

**Table 1:** Published cases of infection caused by *Nocardia pseudobrasiliensis*.

Ref. no. (year)	Age	Gender	Risk factors	Location	Disseminated disease	Treatment	Surgery	Outcome
Present case (2024)	67	M	End-stage kidney disease, renal transplant on tacrolimus and prednisolone	Biceps brachii	Yes—lungs	MEM, LDZ, CIP, TMP-SMX (substituted for AZM)	Drainage	Resolution of symptoms
[12] (2023)	70s	M	SLE on prednisone	Left knee	No	AMC and levofloxacin	Drainage	Resolution of symptoms
[13] (2023)	72	F	COPD	Pulmonary	Yes	TMP-SMX, CIP	NS	Death
[13] (2023)	74	M	Lung cancer on chemotherapy	Pulmonary	Yes	TMP-SMX, AMK, IMP, LDZ, CRO	NS	Death
[14] (2023)	52	M	Multisystem sarcoidosis on hydroxychloroquine and infliximab	Pulmonary	No	TMP-SMX; IMP, LDZ, CIP	NS	Symptom improvement—Treatment ongoing at time of publication
[15] (2020)	67	F	Thorn-related injury	Right digitus medius	No	TMP-SMX	Drainage	Resolution of symptoms
[16] (2018)	49	M	NS	Right eye	No	AMK, TMP-SMX, CIP + corticosteroids	NS	Resolution of symptoms
[17] (2018)	68	M	Renal transplant on tacrolimus, mycophenolate and prednisone	Pulmonary	No	AZM, MEM, LZD	NS	Resolution of symptoms
[18] (2015)	41	M	Chronic alcoholic hepatic disease on prednisolone	Pulmonary	Yes—eye abscess and periorbital cellulitis	IMP, AMC, TMP-SMX	NS	Death
[8] (2015)	79	M	Myasthenia gravis on prednisolone	Pulmonary	Yes—muscle abscesses	LZD, changed to moxifloxacin	Drainage	Resolution of symptoms
[19] (2013)	37	M	Cardiac transplant on cyclosporine, mycophenolate, prednisone	Pulmonary	No	IMP, CRO, CIP	NS	Resolution of symptoms
[3] (2013)	57	F	Pulmonary MPA on prednisolone	Cutaneous (mycetomas)	No	SAM, AMC	NS	Resolution of symptoms
[10] (2012)	86	M	NS	Left knee	Yes—cutaneous abscesses	TMP-SMX, CIP	NS	Resolution of symptoms
[11] (2010)	55	M	Multiple myeloma with previous allogeneic SCT on cyclosporine, mycophenolate and daclizumab	Pulmonary	Yes—cerebral abscess	Piperacillin-tazobactam and AMK, changed to CIP and gentamicin	NS	Death
[20] (2008)	9	F	NS	Ventriculitis/choroid plexitis	No	TMP-SMX, MEM and AMK, changed to CIP and LZD	Stereotactic decompression	Resolution of symptoms
[21] (2003)	71	M	Metastatic solid organ tumour on chemotherapy	Pulmonary	No	NS	NS	NS
[22] (1999)	33	M	HIV/AIDS	Pulmonary	Yes—cutaneous and septic arthritis	Clindamycin, AMK, IMP, TMP-SMX and amphotericin B	NS	Death

*Note:* AMC, amoxicillin-clavulanic acid; AMK, amikacin; AZM, azithromycin; CIP, ciprofloxacin; CRO, ceftriaxone; IMP, imipenem; LZD, linezolid; MEM, meropenem; MPA, microscopic polyangiitis; SAM, sulbactam/ampicillin; TMP-SMX, Trimethoprim/sulfamethoxazole.

Abbreviations: HIV, human immunodeficiency virus; NS, not specified; SCT, stem cell transplant.

**Table 2:** Published cases of nocardiosis with intramuscular involvement.

(Ref. no.) (year)	Age	Gender	Risk factors	Immunosuppressant	Organism	Muscle site affected	Other organs affected	Treatment	Surgery	Outcome
Present case (2024)	67	M	End-stage kidney disease, renal transplant	Tacrolimus and prednisolone	*N. pseudobrasiliensis*	Biceps brachii	Lungs	MEM, LDZ, CIP, TMP-SMX (substituted for AZM)	Drainage	Resolution of symptoms
[23] (2023)	78	F	Myasthenia gravis	Prednisone and mycophenolate	*N. farcinica*	Shoulder	Lungs, bursa, joints	MEM, TMP-SMX (replaced with AMC)	Debridement	Improvement of symptoms but transferred to hospice care
[24] (2022)	57	M	Liver transplant and granulomatosis with polyangiitis	Tacrolimus, methylprednisolone, prednisone	*N. africana*	Thigh	Brain	CRO, TMP-SMX, minocycline	Drainage	Symptom improvement—Treatment ongoing at time of publication
[24] (2022)	61	M	Ischaemic cardiomyopathy, hypertension, bladder cancer and chronic renal failure	Methylprednisolone, prednisolone, mycophenolate	*N. farcinica*	Biceps brachii	Lungs	TMP-SMX (switched to LDZ)	NS	Resolution of symptoms
[1] (2021)	59	M	Renal transplant	Tacrolimus, mycophenolic acid, prednisone and rituximab	*N. paucivorans*	Posterior thigh	Lungs (nodule not confirmed to be nocardiosis)	TMP-SMX and IMP (switched to CRO)	Operative irrigation and debridement	Resolution of symptoms
[25] (2021)	37	M	Minor cutaneous trauma	None	*N. farcinica*	Forearm	N/A	SAM, changed to TMP-SMX	Drainage and debridement	Resolution of symptoms
[26] (2020)	73	M	Not immune-related	None	*N. arthritidis*	Lower leg	Brain, lungs	IMP, TMP-SMX	NS	Symptom improvement
[27] (2020)	65	M	Membranous nephropathy	Cyclophosphamide and prednisone	*N. cyriacigeorgica*	Rhomboid	Brain, lungs	TMP-SMX, LZD, methylprednisolone, MEM	Drainage and ulcer repair	Resolution of symptoms
[28] (2020)	52	M	Dermatomyositis	Prednisone	*Not specified*	Thigh muscle	Skin, joints	TMP-SMX	NS	Resolution of symptoms
[29] (2019)	77	M	Cryptogenic pneumonia	Methylprednisolone	*N. cyriacigeorgica*	Iliacus, gluteal, obturator	Prostate, femoral veins	IMP, AMK and TMP-SMX	Decompression, fasciotomy, drainage	Symptom improvement—Treatment ongoing at time of publication
[30] (2018)	64	M	CKD and heart transplant	Tacrolimus, prednisone and mycophenolate mofetil	*N. asteroides*	Vastus lateralis	Lungs, heart, brain	TMP-SMX (changed to LDZ), IMP–cilastatin (replaced with CRO)	NS	Resolution of symptoms
[31] (2016)	84	M	Polymyalgia rheumatica	Corticosteroid (unspecified)	N/A	Thigh	N/A	Antibiotics (unspecified)	NS	Resolution of symptoms
[8] (2015)	79	M	Myasthenia gravis	Corticosteroid (unspecified)	*N. pseudobrasiliensis*	Thigh	Lungs	LZD, changed to moxifloxacin	Drainage	Resolution of symptoms
[4] (2012)	59	F	Autoimmune haemolytic anaemia	Prednisolone	*N. farcinica*	Thigh	Lung, brain	TMP-SMX, IMP, AMK	Drainage	Resolution of symptoms
[32] (2011)	61	F	Lupus nephritis	Prednisolone	*N. farcinica*	Psoas	Lungs	TMP-SMX, changed to CRO and CIP	Drainage	Resolution of symptoms
[33] (2008)	32	M	AIDS	None	*N. asteroides*	Psoas, paravertebral, maximus gluteus	Bone, lung	AMK, IMP and TMP-SMX (substituted for CIP)	Drainage	Symptom improvement—Treatment ongoing at time of publication
[34] (2007)	46	M	Not immune-related	NS	*N. asteroides*	Iliopsoas	Vertebrae	AMK and IMP (substituted for LDZ)	Drainage	Resolution of symptoms
[35] (2007)	22	F	SLE Nephritis	Prednisone and cyclophosphamide	*N. farcinica*	Paraspinal	Lungs, heart, kidneys, brain	AMK, IMP, TMP-SMX, CIP	NS	Symptom improvement—Treatment ongoing at time of publication
[36] (2007)	76	M	Polymyalgia rheumatica or giant-cell arteritis	Prednisolone	*N. farcinica*	Iliac fossa	Brain, lung	TMP-SMX	Debridement	Resolution of symptoms
[37] (2006)	65	F	Hodgkin's disease	Chemotherapy	*N. farcinica*	Thigh	N/A	TMP-SMX	Drainage	Resolution of symptoms
[38] (2003)	42	M	None	NS	*N. farcinica*	Psoas	N/A	TMP-SMX	Drainage	Resolution of symptoms
[39] (2001)	58	M	Cardiac transplantation	Corticosteroid (unspecified), cyclosporine and azathioprine	*N. asteroides*	Thigh adductor	Lungs	Clarithromycin, CIP, ethambutol, changed to TMP-SMX	Drainage	Resolution of symptoms
[40] (1973)	39	M	NS	NS	*N. brasiliensis*	Thigh	CNS, lungs, spleen	Penicillin and sulfonamides	Drainage	Death

*Note:* AMC, amoxicillin-clavulanic acid; AMK, amikacin; AZM, azithromycin; CIP, ciprofloxacin; CRO, ceftriaxone; IMP, imipenem; LZD, linezolid; MEM, meropenem; SAM, sulbactam/ampicillin; TMP-SMX, Trimethoprim/sulfamethoxazole.

Abbreviation: NS, not specified.
